# Development and application of a machine learning algorithm for classification of elasmobranch behaviour from accelerometry data

**DOI:** 10.1007/s00227-018-3318-y

**Published:** 2018-03-08

**Authors:** L. R. Brewster, J. J. Dale, T. L. Guttridge, S. H. Gruber, A. C. Hansell, M. Elliott, I. G. Cowx, N. M. Whitney, A. C. Gleiss

**Affiliations:** 1 Bimini Biological Field Station Foundation, South Bimini, Bahamas; 2 Institute of Estuarine and Coastal Studies, University of Hull, Hull, HU6 7RX UK; 3 Hull International Fisheries Institute, University of Hull, Hull, HU6 7RX UK; 4 Department of Biology, Hopkins Marine Station, Stanford University, Pacific Grove, CA 93950 USA; 5 Division of Marine Biology and Fisheries, Rosenstiel School of Marine and Atmospheric Science, 4600 Rickenbacker Causeway, Miami, FL 33149 USA; 6 Department of Fisheries Oceanography, School for Marine Science and Technology, University of Massachusetts Dartmouth, 836 South Rodney French Blvd, New Bedford, MA 02719 USA; 7 Anderson Cabot Center for Ocean Life, New England Aquarium, Central Wharf, Boston, MA 02110 USA; 8 Centre For Fish and Fisheries Research, School of Veterinary and Life Sciences, Murdoch University, 90 South Street, Perth, WA 6150 Australia

## Abstract

Discerning behaviours of free-ranging animals allows for quantification of their activity budget, providing important insight into ecology. Over recent years, accelerometers have been used to unveil the cryptic lives of animals. The increased ability of accelerometers to store large quantities of high resolution data has prompted a need for automated behavioural classification. We assessed the performance of several machine learning (ML) classifiers to discern five behaviours performed by accelerometer-equipped juvenile lemon sharks (*Negaprion brevirostris*) at Bimini, Bahamas (25°44′N, 79°16′W). The sharks were observed to exhibit chafing, burst swimming, headshaking, resting and swimming in a semi-captive environment and these observations were used to ground-truth data for ML training and testing. ML methods included logistic regression, an artificial neural network, two random forest models, a gradient boosting model and a voting ensemble (VE) model, which combined the predictions of all other (base) models to improve classifier performance. The macro-averaged *F*-measure, an indicator of classifier performance, showed that the VE model improved overall classification (*F*-measure 0.88) above the strongest base learner model, gradient boosting (0.86). To test whether the VE model provided biologically meaningful results when applied to accelerometer data obtained from wild sharks, we investigated headshaking behaviour, as a proxy for prey capture, in relation to the variables: time of day, tidal phase and season. All variables were significant in predicting prey capture, with predations most likely to occur during early evening and less frequently during the dry season and high tides. These findings support previous hypotheses from sporadic visual observations.

**Electronic supplementary material:**

The online version of this article (10.1007/s00227-018-3318-y) contains supplementary material, which is available to authorized users.

## Introduction

Selecting the optimal behavioural response can increase individual fitness, have adaptive significance and evolutionary consequences (Lima and Dill 1990; McNamara and Houston 1996; Shepard et al. 2008b). Identification of these behaviours as well as their subsequent energetic costs can provide insight into an individual’s activity budget and ecology (Cooke et al. 2004; Metcalfe et al. 2016), in turn impacting populations (Forsman 2015). Identifying and understanding natural behaviours of free-ranging animals is particularly challenging for species living in aquatic environments, because continuous direct observations are impossible to gather (Gleiss et al. 2011a; Brown et al. 2013). If marine animals are to be observed in their natural environment, new techniques need to be developed to monitor them over long periods, in poor visibility (e.g., low light levels or turbid water), at deeper depths and in adverse environmental conditions. Biotelemetry (transmitted biological data) and biologging tools (archival tags; see Cooke 2008 for further details) capable of overcoming such obstacles are now widely available (Cooke et al. 2004; Rutz and Hays 2009; Bograd et al. 2010). One such tool is the acceleration data logger (ADL), a device that measures changes in velocity and can be used to determine body orientation and kinematics for behavioural classification (Shepard et al. 2008b; Sakamoto et al. 2009; Gleiss et al. 2011b; Brown et al. 2013). Data are stored in the device’s on-board memory. These ADLs must be retrieved to obtain data, but allow data to be recorded at higher frequencies, providing insight into fine-scale behaviour. Their application has become increasingly popular for use on animals occupying media that preclude direct observations, and now many ADLs are coupled with additional sensors for monitoring abiotic factors (e.g., temperature and depth, Watanabe et al. 2012; Wright et al. 2014; Lear et al. 2017; Carroll et al. 2014).

Modern ADLs collect large quantities of high resolution acceleration and abiotic data, making deciphering behaviours from acceleration data manually, as was done initially, impractical. This has prompted a need for more automated behaviour classification (Shepard et al. 2008a; Tanha et al. 2012; Bidder et al. 2014), through machine-learning algorithms and the development of new software (Sakamoto et al. 2009; Walker et al. 2015). Machine learning (ML) can be broadly categorised into supervised and unsupervised (Hastie et al. 2009; Valletta et al. 2017), both of which have strengths and weaknesses. In supervised learning, a training set is required whereby the input (e.g., acceleration features) and associated outcome measure/label (e.g., behaviour) are known. Once the input variables can be appropriately mapped to the outcome, the algorithm can be used to make predictions from new input data (Hastie et al. 2009). Examples of these techniques include decision trees, random forest (RF), K-nearest neighbour and linear discriminant analysis (Kiani et al. 1998; Staudenmayer et al. 2009; Nathan et al. 2012; Soltis et al. 2012; Campbell et al. 2013; Bidder et al. 2014; Resheff et al. 2014; Williams et al. 2015; Sur et al. 2017). Supervised ML has been applied to classify acceleration data in many studies and has the advantage of clearly defined behaviours and simple interpretation (Leos-Barajas et al. 2017). However, it demands a comprehensive training data set which can be unattainable for some species and requires a validation process (Allen et al. 2016). Selection of the optimum supervised ML method can also be time-consuming (Ladds et al. 2017). Clustering algorithms such as *k*-means clustering and principal component analysis, where no outcome measure is provided, are examples of unsupervised learning methods (Sakamoto et al. 2009; Valletta et al. 2017). The algorithm groups data based on inherent similarities between input variables (Hastie et al. 2009). Unsupervised learning has the potential to reveal novel behavioural patterns (Battaile et al. 2015; Sakamoto et al. 2009; Chimienti et al. 2016) and is particularly valuable for species that are not readily adaptable in captivity or are not easily observed in the wild, hindering direct observation during data collection (i.e., ground-truthing). However, in the case of *k*-means clustering, drawbacks include a priori specification of the number of behaviours reflected in the dataset by setting the number of clusters. The optimum number of clusters can often be ambiguous with too few clusters resulting in similar behaviours being grouped together, whilst too many may artificially separate behaviours (Sakamoto et al. 2009; Whitney et al. 2010; Gleiss et al. 2017; Valletta et al. 2017). Some ML algorithms such as artificial neural networks (ANN) and hidden Markov models can be used in both a supervised and unsupervised learning context (Schmidhuber 2015; Leos-Barajas et al. 2017).

 Ensemble classifiers combine the strengths of multiple supervised machine learners (base learners), to improve overall prediction accuracy of the model (ratio of correct predictions over number of total predictions, Hastie et al. 2009). Ladds et al. (2017) classified acceleration data obtained on captive fur seals and sea lions into behavioural categories using super learning—a form of ensemble learning—whereby the output of the base learners is used as additional data to inform the super-learning algorithm. Their optimum model achieved superior accuracy (85.1% for four behavioural categories) and lower variance than any of the constituent models. Dutta et al. (2015) tested three ensemble classifier techniques: Adaboost, Random Subspace and bagging, finding the latter could achieve 96% accuracy in classifying acceleration data from cattle into five classes. Voting ensemble classifiers are one of the simplest to implement and the decision rule can be based on the majority vote, averaging probabilities or product of probabilities (see Catal et al. 2015 for comparison of results between methods). However, we are not aware of the application of this form of ensemble classifier to acceleration data obtained on non-humans or of ensemble classifiers for predicting behavioural states for animals at liberty.

Whilst ML techniques have been applied to classify acceleration data obtained from a variety of terrestrial fauna (e.g., vultures, Nathan et al. 2012; cheetahs, Grünewälder et al. 2012; badgers, McClune et al. 2014; pumas, Wang et al. 2015; cows, Martiskainen et al. 2009; Diosdado et al. 2015; condors, Williams et al. 2015) and some air-breathing marine fauna (e.g., cetaceans, Allen et al. 2016; Owen et al. 2016; pinnipeds, Battaile et al. 2015; Ladds et al. 2017; and penguins, Yoda et al. 1999; Carroll et al. 2014; Chessa et al. 2017), their use on elasmobranch acceleration data has been limited to two unsupervised methods (Whitney et al. 2010; Leos-Barajas et al. 2017). Sharks regularly occupy high trophic positions (Cortés 1999; Estrada et al. 2003) and can influence the structure of marine ecosystems (Heithaus et al. 2008; Rasher et al. 2017; Barley et al. 2017). However, their typically high mobility and inaccessible habitat make their natural behaviour difficult or impossible to observe directly (Klimley et al. 1992; Nakamura et al. 2011; Watanabe et al. 2012; Payne et al. 2016). To date, accelerometer application with sharks has provided new information on activity patterns (Whitney et al. 2007; Gleiss et al. 2013; Leos-Barajas et al. 2017; Gleiss et al. 2017), mating behaviour (Whitney et al. 2010), metabolic demands (Gleiss et al. 2010; Barnett et al. 2016; Whitney et al. 2016b; Bouyoucos et al. 2017; Lear et al. 2017), post-release mortality (Whitney et al. 2016a) and biomechanics (Gleiss et al. 2011a; Payne et al. 2016; Papastamatiou et al. 2018). However, in sharks, behavioural classification has relied upon visual inspection of the data or unsupervised ML methods (Whitney et al. 2010; Leos-Barajas et al. 2017), where overall classification performance cannot be quantified.

Despite the above applications of ADLs to study sharks, this technology has not yet been used to investigate their feeding behaviour. Many sharks are thought to be predominantly opportunistic, asynchronous feeders (Wetherbee et al. 1990; Newman et al. 2010). Knowledge of the feeding ecology of a species, including feeding frequency and periodicity, is required for developing ecosystem models and predicting the impact of population decline (Stevens et al. 2000). Through stomach eversions and digestion analysis, Bush (2003) showed that juvenile scalloped hammerhead sharks (*Sphyrna lewini*) are nocturnal hunters. Higher quantities of food were also found in their stomachs during the winter, but this could be the result of slower gastric evacuation at reduced temperatures rather than an increase in consumption. Accelerometers may provide a simpler, more accurate and less invasive method for investigating feeding frequency and periodicity than stomach content analysis and allow identification of behaviours for a single animal over extended time periods rather than single point measures.

Here a tool is developed to classify shark behaviour from accelerometry data, using the juvenile lemon shark (*Negaprion brevirostris*) as a model species due to its hardiness in captivity, its abundance, and high site fidelity to nursery grounds at the study site, Bimini, Bahamas (Gruber 1982; Morrissey and Gruber 1993). This study used ADLs, semi-captive behavioural observations and ML algorithms to accomplish three objectives: (1) obtain ground-truthed behavioural observations of accelerometer-equipped lemon sharks; (2) use ground-truthed data to generate and assess the performance of supervised ML algorithms to predict wild lemon shark behaviour, and (3) explore the applicability of these predictions in relation to abiotic factors to gain insight into the behavioural ecology of the juvenile lemon shark.

## Materials and methods

### Tag package

The tag package consisted of a triaxial acceleration data logger (G6a+ ADL; 40 mm × 28 mm × 17 mm, 30 Hz, 56 MB, CEFAS Technologies Ltd) coupled, using epoxy resin, with an acoustic transmitter (Sonotronics PT-4; 9 mm × 25 mm, 134–136 dB, battery life: 3 months, Sonotronics Inc) for tag retrieval via acoustic telemetry. It was attached to individuals by puncturing two holes (1.5 mm diameter), using a hypodermic needle, through the base of the first dorsal fin and passing nylon monofilament through the ADL and the fin (Sundström et al. 2001; Gleiss et al. 2009a; Lear et al. 2017). On the opposite side of the dorsal fin, the ends of the monofilament were secured against two small plastic plates using stainless steel crimps. A medical-grade polyurethane foam (Poron Blue Medical Grade 4708, 1.5 mm thick, unabraded) was placed between the plastic plates and the ADL to minimise rubbing and skin damage.

### Captive trials

This study was conducted around the Bimini Islands, Bahamas (25°44′N, 79°16′W), two small mangrove fringed islands approximately 85 km due east of Miami, Florida, USA. The system has been extensively studied by the Bimini Biological Field Station Foundation and provides well-documented nursery grounds for juvenile lemon sharks (Chapman et al. 2009). For captive trials, juvenile lemon sharks were caught (*n* = 4) using a 180 m × 2 m monofilament gillnet set perpendicular to the shoreline of South Bimini (Gruber et al. 2001; Table 1). To reduce the risk of mortality, nets were checked at 15-min intervals or when a disturbance was detected. Previously captured individuals were identified using an intramuscular passive integrated transponder (PIT; Destron Fearing Inc.; Gruber et al. 2001) injected under the skin at the base of the dorsal fin. Sharks of appropriate size [75–90 cm total length (TL)] were transported to a nearby rectangular semi-enclosed pen (10 × 6 m) erected on the neighbouring sand flats. The minimum TL was dictated by the size and mass of the tag package, whilst the maximum TL represented the largest animals that could be housed in a respirometer for a separate component of a larger study (Lear et al. 2017). A large rectangular pen was used to minimise the repetitive circular swimming patterns previously observed when housing juvenile lemon sharks in a circular pen (Gleiss et al. 2009a). The pens were constructed from plastic diamond mesh, allowing the sharks to experience natural ambient conditions (i.e., salinity, temperature, tides, lunar cycles; Guttridge 2009). Except during trials, animals were fed to satiation with thawed or fresh fish every third day (Wetherbee et al. 1987; Cortés and Gruber 1994; Guttridge et al. 2009). To allow sharks the opportunity to recover and to acclimatize to the pen, ADL packages were attached after a period of at least 2 days within the pen. Behavioural trials to develop an ethogram (i.e., a catalogue of distinct activities constituting the behavioural repertoire of an animal; Grier 1984; Sakamoto et al. 2009) began ~ 24-h after ADL attachment.

**Table 1:** Juvenile lemon sharks that exhibited the five behaviours for classification during semi-captive trials for development of an acceleration ethogram

PIT tag ID	Sex	Total length (cm)	Weight (kg)
985121031792723	Female	82.6	3.75
4C4A2D3A12	Female	80.5	3.10
4C3A6C313A	Male	79.2	3.15
4C3B312275	Male	85.2	3.75

To view captive sharks, a wooden tower (height 3 m) was placed next to the pen. Observers recorded behaviour/body movements to the second using digital clocks synchronized with the ADL. These observations were paired with the acceleration measurements to be used in developing a classification algorithm for predicting behaviour from individuals at liberty. Juvenile lemon sharks have been observed swimming, chafing (flashing), resting, burst-swimming and feeding around the Bimini Islands. Therefore, we focused on obtaining acceleration signatures for these behaviours during captive trials. Burst events performed in the wild were witnessed in response to disturbances (e.g., passing boats), predators, and during hunting. Successful prey capture in the wild was accompanied by side-to-side headshakes (authors’ pers. obs). Chafing, a behaviour hypothesised as a method for dislodging parasites (Myrberg Jr and Gruber 1974), was characterised by a roll motion whereby the dorsal surface of the shark came into contact with the substrate or water surface. Resting behaviour was defined as individuals lying motionless on the seabed. To train the classifiers, more replicates of rare behaviours were needed than were readily displayed in the pens and subsequently some behaviours were induced. For example, burst events were prompted by throwing dive weights to the side or behind an individual or by making large movements next to the pen. Following completion of the ethogram trial, the shark was recaptured using a dip-net and the ADL package was removed. Individuals were monitored for several days prior to release.

### Data collection from free-ranging lemon sharks

Juvenile lemon sharks are known to frequent a mangrove inlet at Bimini, Bahamas on a daily basis and being a shallow and sheltered area it provides opportunity to deploy ADLs and conduct observations of wild shark behaviours (Guttridge 2009; Guttridge et al. 2012). Individuals were captured using a dip-net as they exited the mangrove inlet on the ebbing tide. They were transported to a small research vessel anchored several metres away from the entrance to the inlet, and placed in a circular tub (1 m diameter) for tag attachment and collection of morphometric data (i.e., TL (cm), weight (kg) and sex; Table 2). All individuals were scanned for presence of a PIT tag and newly captured sharks had a PIT tag inserted. Following tag attachment, the shark was manually carried from the research vessel and released in the direction to which it was heading prior to capture. The duration of capture to release was ~ 5 min. ADLs were attached approximately 24 h prior to the commencement of data logging and recorded data (acceleration 30 Hz; pressure and temperature 1 Hz) for 120 h. A 24-h delay of the commencement of logging by the tag allowed for post-release recovery, increasing the chances to record normal behaviour.

**Table 2:** Wild lemon sharks tagged with the accelerometer data logger/acoustic transmitter package. Seasons are split into wet (April–September; *n* = 10) and dry season (October–March; *n* = 10)

Pit tag#	Season	Sex	Dates	Total length (TL) (cm)	Weight (kg)
4A0A043D40^a^	Wet	F	29/07/12–03/08/12	77.5	3.25
4A73536511	Wet	F	29/07/12–03/08/12	83.6	3.00
4A66401437	Wet	F	29/07/12–03/08/12	78.1	2.80
4A44545C6C	Wet	M	31/08/12–05/09/12	82.4	3.25
4A63380105^b^	Wet	F	31/08/12–05/09/12	81.4	2.75
4C3B211816	Wet	F	31/08/12–05/09/12	81.0	2.10
4B7B473332	Wet	F	31/08/12–05/09/12	83.1	3.40
4A68061232	Wet	M	31/08/12–05/09/12	74.3	2.60
4C3B086000	Wet	M	31/08/12–05/09/12	76.0	2.10
985121031823859	Wet	F	29/08/14–03/09/14	77.5	2.30
4B7B442028	Dry	M	12/01/13–17/01/13	80.6	2.75
4A63380105^b^	Dry	F	12/01/13–17/01/13	82.5	3.50
4A603C232D	Dry	M	12/01/13–17/01/13	87.3	3.25
4C3B2A712D	Dry	F	26/03/14–31/03/14	88.3	3.17
4A0A043D40^a^	Dry	F	26/03/14–31/03/14	89.0	4.10
4C3B032B0C	Dry	F	26/03/14–31/03/14	86.5	3.10
4A5A577669	Dry	M	08/11/14–13/11/14	88.4	3.60
4B7B464873	Dry	M	08/11/14–13/11/14	87.1	3.50
4C497D6463	Dry	F	08/11/14–13/11/14	78.4	2.40
4C4A736341	Dry	F	08/11/14–13/11/14	81.7	3.00

^a,b^Indicate individuals tagged during both seasons

Various techniques were employed to recapture tagged individuals: the mangrove inlet was seined off over high tides as per the capture process; stationary baited gillnets were placed across the flats at various locations and checked every 15 min or when there was a disturbance, whilst boats with acoustic tracking gear scanned the area listening for PT-4 transmitters. Sharks located by acoustic telemetry would be encircled with a gillnet, and the tags were removed upon capture. The ADL attachment site healed in < 30 days with recaptured animals displaying no apparent marks in subsequent seasons.

### Data analysis

**Fig. 1:**
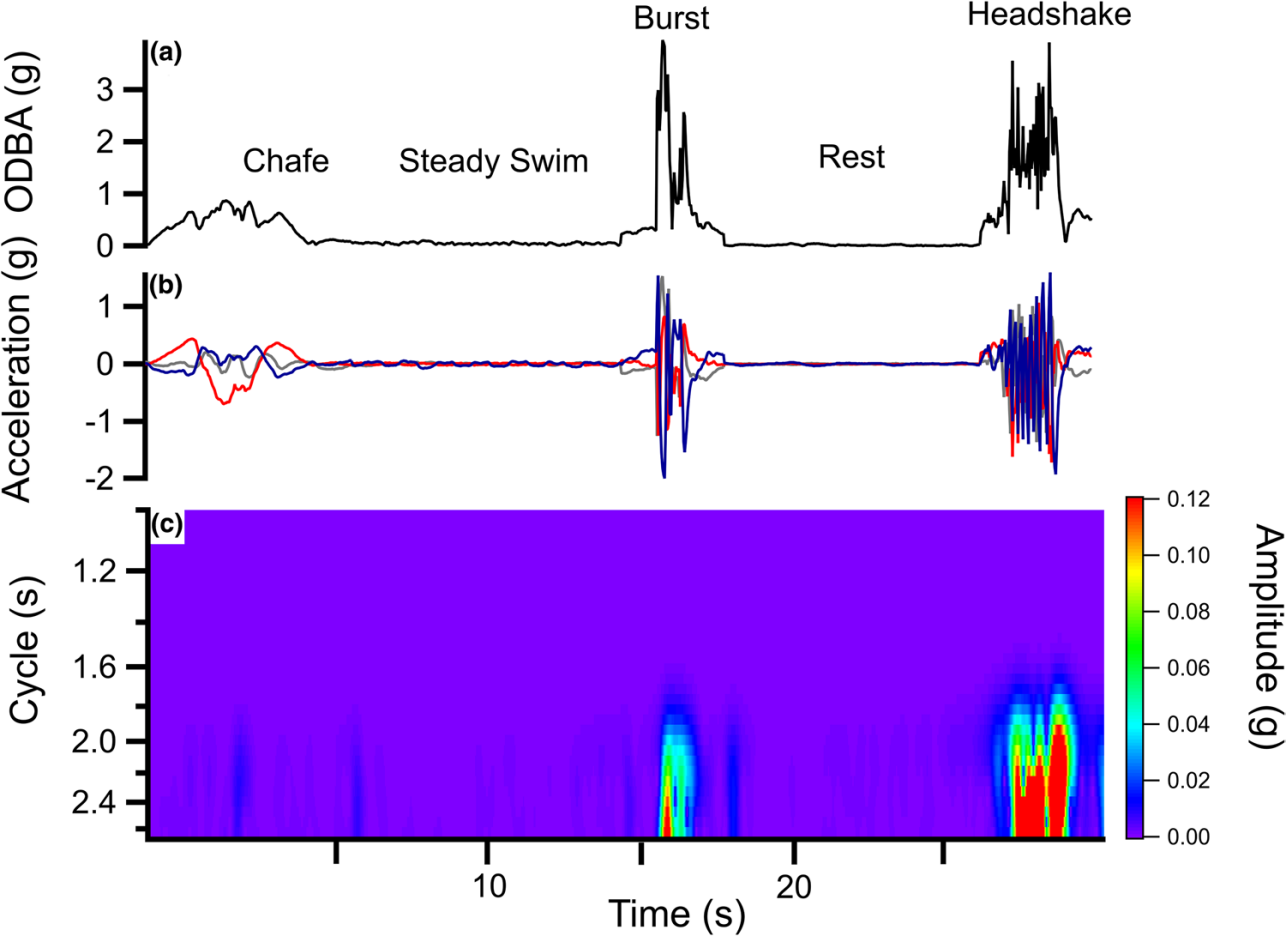
**a** Examples of the five behaviours for classification. Overall dynamic body acceleration (ODBA) is calculated as the sum of the absolute values of dynamic acceleration from the three axes. **b** Dynamic acceleration in the three orthogonal axes: sway (blue), heave (red) and surge (grey) during each behaviour and **c** corresponding wavelet spectrum generated from the sway axis showing increased signal strength amplitude during the burst and headshake event

ADL data analysis was conducted in Igor Pro version 6.34 (WaveMetrics Inc, Lake Oswego, Oregon, USA). Static acceleration, which measures the orientation of the accelerometer in relation to the earth’s gravitational pull, representing animal posture, was extracted from the acceleration data (as recorded by the ADL) using 3 s box smoothing (Shepard et al. 2008a). After separation, dynamic acceleration, a measure of the animals’ movement, remained for each orthogonal axis: surge, heave and sway (x, y, z; Fig. 1). Surge denotes anterior–posterior movement, heave represents dorsal–ventral movement and sway is lateral movement. Typical routine swimming is characterised by regular oscillations in the dynamic swaying acceleration of sharks, representing individual tail-beats (Gleiss et al. 2009a, b). Overall dynamic body acceleration (ODBA) was calculated as the sum of the absolute dynamic axes values (Wilson et al. 2006). The signal strength amplitude and frequency of the dominant cycle from the sway axis was extracted using continuous wavelet transformation, with the Morlet mother wavelet function through Ethographer v2.0 (Sakamoto et al. 2009; available from http://bre.soc.i.kyoto-u.ac.jp/bls/index.php?Ethographer; Fig. 1). These feature vectors form part of the acceleration summary statistics calculated for use as predictor variables in this multi-class classification scenario (Table 3; Nathan et al. 2012; Zheng et al. 2013; Wang et al. 2015).

**Table 3:** Features extracted from acceleration data loggers and used to train the base learner classifiers (see Zheng et al. 2013 for equations)

Parameter	Label	Definition
Static acceleration	Xstat, Ystat, Zstat	Static acceleration for each axis reflective of body orientation
Dynamic acceleration	Xdyn, Ydyn, Zdyn	1 s means of body movement generated acceleration in each axis
Overall Dynamic Body Acceleration	ODBA	Sum of the absolute values from the three dynamic axis
Amplitude	Amp	Amplitude of the signal derived from the sway axis body movement
Frequency	Hz	Dominant tailbeat frequency from lateral acceleration
Standard deviation	XstatSD, YstatSD, ZstatSD, XdynSD, YdynSD, ZdynSD, ODBASD	Standard deviation of static and dynamic acceleration measures in each axis
Skewness	XstatSkew, YstatSkew, ZstatSkew, XdynSkew, YdynSkew, ZdynSkew, ODBASkew	A measure of the symmetry of the feature vector
Kurtosis	XstatKurt, YstatKurt, ZstatKurt, XdynKurt, YdynKurt, ZdynKurt, ODBAKurt	A measure of the tail shape of the feature vector
Maximum	XstatMax, YstatMax, ZstatMax, XdynMax, YdynMax, ZdynMax, ODBAMax	Maximum values per second for dynamic and static acceleration in each axis and for ODBA
Minimum	XstatMin, YstatMin, ZstatMin, XdynMin, YdynMin, ZdynMin, ODBAMin	Minimum values per second for dynamic and static acceleration in each axis and for ODBA
Frequencies from wavelet spectra	X.values	Amplitude for the relevant frequency obtained through the continuous wavelet transformation generated spectrogram

Static acceleration was calculated from the raw acceleration using 3-s box smoothing, leaving dynamic acceleration remaining. Overall dynamic body acceleration (ODBA) is calculated as the sum of the absolute values of dynamic acceleration from the three axes

#### Classifier development

To classify the behaviour of wild sharks into the five behavioural categories established during the captive ethogram trials, an ensemble classifier model was built using ML base models from the ‘scikit-learn' package (Pedregosa et al. 2011) in Python (Python Software Foundation, Python Language Reference, version 2.7; available at http://www.python.org). The ground-truthed data were split into three portions: (1) the training set (60%) for developing all base learner models; (2) the validation set (20%) for model selection as well as weighting; and (3) the test set (20%) to estimate the generalisation error and overall performance of the selected final model (Hastie et al. 2009). The data splits were randomized and implemented using stratified sampling in the ‘scikit-learn' package to preserve the relative class frequencies in each data set. For each observation, base models generated the probability that the observation belonged to each class. As such, only classification models capable of generating probabilities (rather than only class labels) were considered for the ensemble classifier built here (henceforth referred to as voting ensemble; VE). The array of probabilities predicted for each observation and class were then averaged across all of the models selected during the validation stage, and the class with the highest predicted probability was selected as the final predicted class value.

The best performing base learners, as established from confusion matrices, were selected for the VE using the validation set. They include logistic regression (LR), a multilayer perceptron artificial neural network (ANN), two random forest (RF) models and a gradient tree boosting (GB) model. The following section provides a brief overview of the theory underlying each base learner model selected.

The LR model used a ‘one-vs-all’ technique. This reduces a multiclass scenario into multiple binary ones, where a logistic model is created for each class versus all remaining classes (Rifkin and Klautau 2004). For new data, each model provides a probability estimate of an observation, with the observation being assigned to the class with the highest probability score.

ANNs are a non-linear regression or classification technique used to model the relationship between predictors and a response variable (Staudenmayer et al. 2009). Multilayer perceptrons are feed-forward ANNs. Input data are mapped onto known output nodes/classes in the final layer, through hidden layers of nodes using a non-linear activation function. In this instance, one hidden layer was implemented with 100 nodes. Each node is connected to nodes in the subsequent layer, but with different connection weights reflecting the importance of the connections. At first, these weights are randomly assigned. The predicted output is compared to the known output and the error between them is passed backwards through the layers, adjusting the connection weights between nodes accordingly. This is known as backpropagation and is a process that is repeated until the model error is deemed to be at an acceptable level. The softmax function, a generalized logistic function, is applied as the output layer to allow for multi-class classification with probability estimates.

RF analysis is a leading ML algorithm that has been applied successfully to accelerometer data for behaviour recognition in a variety of species (Casale et al. 2011; Graf et al. 2015; Luštrek and Kaluža 2009; Nathan et al. 2012; Wang et al. 2015). RF is a ‘supervised ensemble classifier’ in itself, whereby many un-pruned classification trees are generated, with each tree voting for a class. RF incorporates two levels of randomness to minimise overfitting: (1) a bootstrap sample of data (62.3%) are used to generate every tree and (2) at each tree node, a subset of predictor variables (m) is selected at random to encourage tree diversity. The remaining data, not used in the bootstrap sample, are used to determine the misclassification rate (Breiman et al. 1984). In most cases, the prediction is made by majority vote from all trees within ‘the forest’, however, the ‘scikit-learn' implementation averages the probabilistic prediction from each classifier to generate a final prediction. For the VE, two RF models were generated from the training data, differing by the split criterion used for choosing the best splitting attribute at each node, i.e., the Gini impurity (model referred to forthwith as RFG), and entropy (RFE) which measures information gained and is most commonly used in classification scenarios.

GB is another ensemble learning method whereby a forward stage-wise additive model is built (Friedman 2001). Unlike RF where each tree is grown extensively, in GB, the trees are very shallow (e.g., they may only have one spilt and is then termed a ‘decision stump’). Weak decision trees are iteratively built, optimising the parameters of the most recent tree, whilst maintaining the parameters of earlier trees to reduce over-fitting. Subsequent trees focus on earlier incorrect predictions, trying to correct those and minimise the deviance loss function (Hastie et al. 2009). In this study, 100 base learner decision trees were fitted with a maximum tree depth of three.

Whilst the predictive power of ANN, GB and RF ML techniques is often improved over simple decision trees, they are commonly referred to as ‘black box’ algorithms, since their decision-making rules are difficult to interpret (Hastie et al. 2009). However, GB and RF do allow for relative ranking of predictor variable importance (Breiman et al. 1984). This allows insight into the features most influencing classification and can be used for variable selection where there are many variables.

#### Evaluation metrics

Metrics calculated from the confusion matrix include precision, recall and the *F*-measure and are commonly used to judge the quality of a classification model (Chen et al. 2004; Özgür et al. 2005). They are calculated from the true positive (TP), false positive (FP) and false negative (FN) values in the confusion matrix. TPs are those that have been correctly assigned to their class, and therefore equal the number in the row and column cell corresponding to the class in question. FPs are those that are incorrectly classified to a class and therefore, in a multiclass classifier, are found by summing the values in the class column, excluding TP. FNs are those that belong to a class but have not been assigned to it and are calculated by summing the values of the class row, excluding the TP. From these values, several indices of performance can be calculated (Özgür et al. 2005) and used to determine the macro-averaged *F*-measure for evaluating overall classification performance. The performances indices are as follows:

Recall: the ratio of correctly identified classes to all known correct classes (Eq. 1):

1 $${\text{Re = }}\frac{{{\text{TP}}_{i} }}{{{\text{TP}}_{i} {\text{ + FN}}_{i} }}.$$

Precision: the fraction of correctly identified classes (i.e., correct recall) against all predicted classes (Eq. 2). A classification model may have good recall for a class if many known observations are correctly identified, but poor precision if this is accompanied by many observations being incorrectly assigned to that class (i.e., a high number of FNs; Sokolova and Lapalme 2009).

2 $${\text{Pr = }}\frac{{{\text{TP}}_{i} }}{{{\text{TP}}_{i} {\text{ + FP}}_{i} }}.$$

*F*-measure: the harmonic mean of recall and precision for each class (Eq. 3).

3 $$F_{i} = \frac{{2\Pr_{i} \text{Re}_{i} }}{{\Pr_{i} + \text{Re}_{i} }}.$$

Macro-average *F*-measure: the mean of the *F*-measures determined for each class (Eq. 4).

4 $$F\left( {\text{macro-averaged}} \right){ = }\frac{{\sum\nolimits_{{i{ = 1}}}^{M} {F_{i} } }}{M}$$

*M*, in Eq. 4 represents the number of classes in the classification problem. Both the *F*-measure and macro-averaged *F*-measure are represented by a value in the range 0–1, with larger values representing improved classification quality. In this study, the optimal model was selected using the macro-averaged *F*-measure. This metric gives equal weight to all classes, regardless of case frequency (Özgür et al. 2005).

#### Classifier application

The VE model was used to predict the behavioural class for each second of ADL data obtained in the wild. A single successful predation event was variable in duration and sometimes incorporated intermittent headshaking. Therefore, the behaviour was considered either present or absent per hour of tag deployment to ensure each predation event contributed equally to the dataset. As all visually observed headshakes in captivity lasted for a minimum of 2 s, headshakes that lasted less than two consecutive seconds were filtered from the data to minimise the impact of false positive readings (Carroll et al. 2014). All hours that included remaining headshakes were subsequently defined as headshaking being present.

Generalized additive mixed models (GAMMs) are a semi-parametric approach used for modelling effects in response to a variety of predictor variables (Hastie and Tibshirani 1990). A GAMM with a binomial distribution was employed to model the absence or presence of headshaking behaviour of juvenile lemon sharks (Table 4).

**Table 4:** Covariates included in binomial generalized additive mixed model investigating headshake events in juvenile lemon sharks in Bimini, Bahamas

Variable	Range	Description	Variable Type
Time of day	0–23 h	24-h day	Cyclic smoother
Season	Dry/wet	Season sharks were tagged	Categorical
Tidal phase	Ebb–Low–Flood–High	Tidal phase based on NOAA’s tidal charts. High and low tide were categorised as one hour either side of event	Categorical
Shark ID	1–20	Influence of individual shark	Random effect

Co-linearity of covariates was investigated using generalized variance-inflation factor (GVIF) scores. Any covariate with a score greater than three was removed and the GVIFs were recalculated (Zuur and Ieno 2012). High and low tidal phases were considered to be 1 h either side of peak high and low tide, with flood and ebb phases occupying the times in between. The tides in the Bimini lagoon and the refuge spot are known to lag approximately 1 h behind those at NOAA’s North Bimini station (ID: TEC4617) and tidal phase was calculated accordingly (Guttridge et al. 2012). Shark ID was incorporated as a random effect to avoid pseudo-replication. The modelling was implemented using the ‘gamm4’ package in R (version 3.3.2). Significance was determined at the 0.05 level. The optimum model was selected using log-likelihood scores, which measure the lack of fit (Johnson and Omland 2004; Wasserman 2000). Scores closest to zero represent optimal fit.

## Results

### Captive trials

**Fig. 2:**
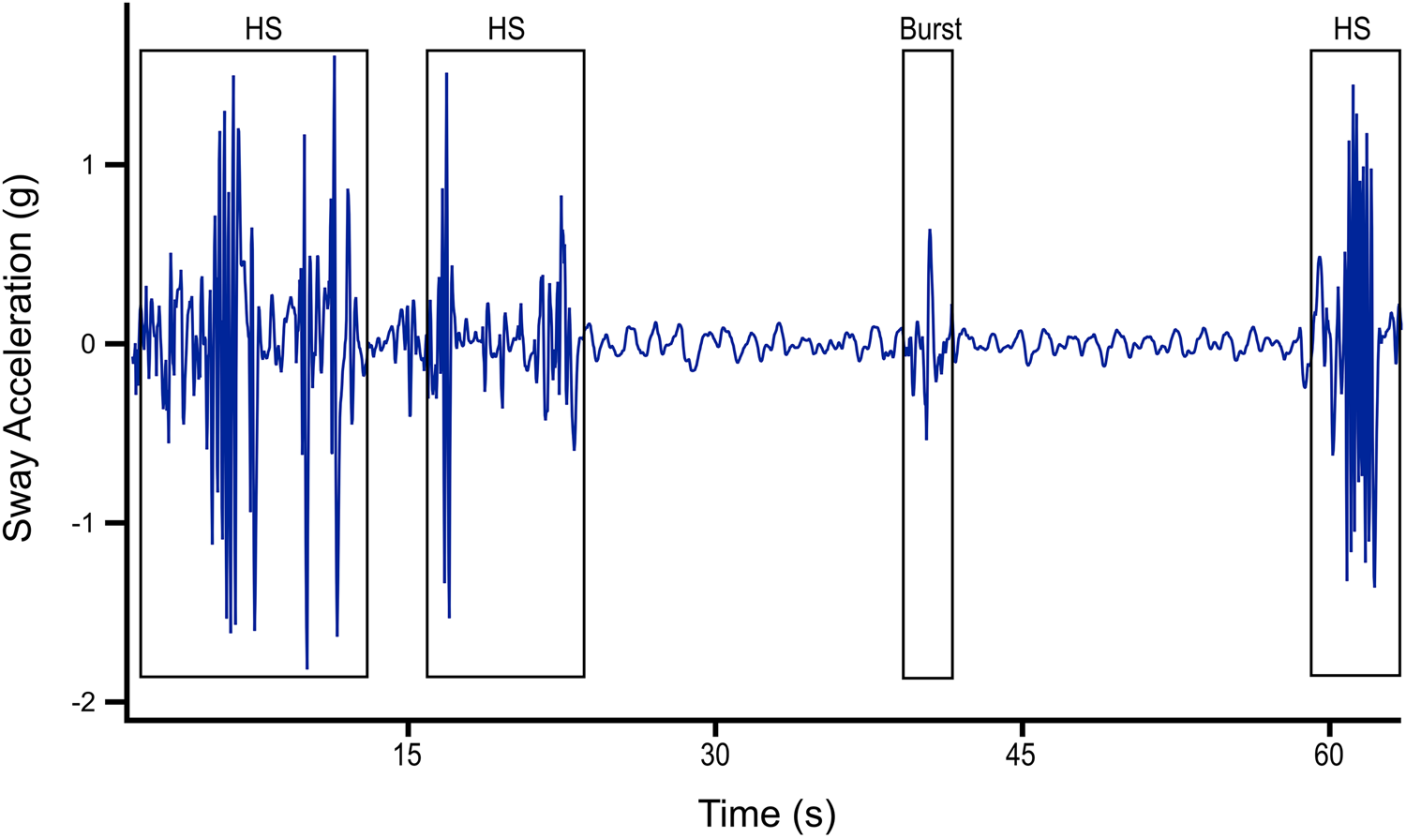
An example from the sway acceleration axis of a 63 s prey manipulation event, consisting of three headshakes (HS; totalling 19 s) and a brief burst event

Resting occurred on six occasions (73.3 ± 107.4 s; range 12–291 s) during captive trials. Chafing behaviour occurred naturally in the pens by individuals with (4.6 ± 1.6 s, *n* = 58; range 3–11 s) and without tag packages. Thirty-five burst events were recorded (1.3 ± 0.5 s; range 1–3 s). Feeding occurred sporadically in the pen and was usually accompanied by side-to-side headshakes, a movement that was not witnessed outside of prey capture. Eight instances of feeding occurred by ADL equipped sharks in the pen, all on their preferred prey species (yellow fin mojarra; *Gerres cinereus*), and seven of which elicited headshaking. The single feeding event that did not result in headshaking consisted of a gulping motion on a smaller fish and was not discernible from swimming behaviour by the dorsally mounted ADL. As such, prey manipulation period was defined here as the duration between the commencement and cessation of headshaking for a single prey item. Successful prey manipulation events varied in duration from 2 to 559 s. Headshaking occurred intermittently for a total of 113 s during prey manipulation. In 50% of instances, the shark did not consume the whole prey item after one headshake, but continued to hold the prey in its mouth or dropped it and displayed further headshaking upon re-collecting it (Fig. 2).

### Model development and performance

The confusion matrices presented in Table 5 were used to calculate the evaluation metrics for all base learners and the VE model (Table 6). As the GB model performed best of all the base learner models during the validation stage, it was weighted three times more than other models in the VE. This marginally improved the macro-averaged *F*-measure by 0.005. The remaining models were weighted once and had to agree confidently in their predictions to override a differing prediction from the GB model. Subsequently, the final VE output is similar to the GB model, with a few erroneous predictions corrected for improved performance. For the swim class, the ANN and GB models provided the optimum recall, with the RF classifiers providing the lowest recall values, but slightly higher precision. The RF and GB models all obtained a precision value of 1 for resting behaviour and obtained the highest recall values ranging from 0.955 to 0.966. LR obtained the lowest value for both precision (0.973) and recall (0.830) in this class. The ANN model provided the poorest recall value for the chafe class but the highest precision (0.957). GB supplied the next highest precision value (0.927) along with the best recall (0.944), whilst the RFG model yielded the worst precision value (0.831).

**Table 5:** Confusion matrix generated for the test set of the ground-truthed data

	Model	Predicted behaviours						
		Class	Swim	HS	Rest	Chafe	Burst	Class error
Actual behaviours	LR	Swim	*6984*	6	2	8	0	0.002
		HS	4	*16*	0	1	2	0.304
		Rest	15	0	*73*	0	0	0.170
		Chafe	3	3	0	*48*	0	0.111
		Burst	0	8	0	0	*2*	0.800
	ANN	Swim	*6995*	2	1	2	0	0.001
		HS	6	*11*	0	0	6	0.522
		Rest	7	0	*81*	0	0	0.080
		Chafe	6	3	0	*44*	1	0.185
		Burst	0	2	0	0	*8*	0.200
	RFG	Swim	*6969*	20	0	9	2	0.004
		HS	1	*19*	0	1	2	0.174
		Rest	3	0	*85*	0	0	0.034
		Chafe	2	2	0	*49*	1	0.093
		Burst	0	3	0	0	*7*	0.300
	RFE	Swim	*6968*	24	0	8	0	0.005
		HS	1	*18*	0	1	3	0.217
		Rest	3	0	*85*	0	0	0.034
		Chafe	2	3	0	*48*	1	0.111
		Burst	0	3	0	0	*7*	0.300
	GB	Swim	*6995*	2	0	3	0	0.001
		HS	5	*16*	0	1	1	0.304
		Rest	4	0	*84*	0	0	0.045
		Chafe	2	0	0	*51*	1	0.056
		Burst	5	0	0	0	*5*	0.500
	VE	Swim	*6995*	2	0	3	0	0.001
		HS	4	*17*	0	1	1	0.261
		Rest	4	0	*84*	0	0	0.045
		Chafe	2	0	0	*51*	1	0.056
		Burst	2	1	0	0	*7*	0.300

Rows indicate actual observations and columns represent predicted behaviours

Values in italic are correctly classified behavioural observations

*LR* logistic regression, *ANN* artificial neural network, *RFG* random forest Gini, *RFE* random forest entropy, *GB* gradient tree boosting, *VE* voting ensemble, *HS* headshakes

**Table 6:** Performance metrics of base learner models and voting ensemble model

Model	Class	TP	FP	FN	Precision	Recall	Class *F*-measure	Macro-averaged *F*-measure
LR	Swim	6984	22	16	0.997	0.998	0.997	0.723
	HS	16	17	7	0.485	0.696	0.571	
	Rest	73	2	15	0.973	0.830	0.896	
	Chafe	48	9	6	0.842	0.889	0.865	
	Burst	2	2	8	0.500	0.200	0.286	
ANN	Swim	6995	19	5	0.997	*0.999*	0.998	0.802
	HS	11	7	12	0.611	0.478	0.537	
	Rest	81	1	7	0.988	0.920	0.953	
	Chafe	44	2	10	*0.957*	0.815	0.880	
	Burst	8	7	2	0.533	*0.800*	0.640	
RFG	Swim	6969	6	31	*0.999*	0.996	0.997	0.810
	HS	19	25	4	0.432	*0.826*	0.567	
	Rest	85	0	3	*1.000*	*0.966*	*0.983*	
	Chafe	49	10	5	0.831	0.907	0.867	
	Burst	7	5	3	0.583	0.700	0.636	
RFE	Swim	6968	6	32	*0.999*	0.995	0.997	0.804
	HS	18	30	5	0.375	0.783	0.507	
	Rest	85	0	3	*1.000*	*0.966*	*0.983*	
	Chafe	48	9	6	0.842	0.889	0.865	
	Burst	7	4	3	0.636	0.700	0.667	
GB	Swim	6995	16	5	0.998	*0.999*	0.999	0.856
	HS	16	2	7	*0.889*	0.696	0.780	
	Rest	84	0	4	*1.000*	0.955	0.977	
	Chafe	51	4	3	0.927	*0.944*	0.936	
	Burst	5	2	5	0.714	0.500	0.588	
VE	Swim	6995	12	5	0.998	*0.999*	*0.999*	*0.888*
	HS	17	3	6	0.850	0.739	*0.791*	
	Rest	84	0	4	*1.000*	0.955	0.977	
	Chafe	51	4	3	0.927	*0.944*	*0.936*	
	Burst	7	2	3	*0.778*	0.700	*0.737*	

The values in italic show optimum values for each metric

*LR* logistic regression, *ANN* artificial neural network, *RFG* random forest Gini, *RFE* random forest entropy, *GB* gradient tree boosting, *VE* voting ensemble, *HS* headshake class; *TP* true positive, *FP* false positive, *FN* false negative

Events from the burst and headshake classes had the highest-class errors (Table 5), yielding the lowest overall *F*-measures of all the classes from the VE (0.737 and 0.791, respectively). The RF models correctly classified the most instances for headshaking behaviour, but the improved recall was at the expense of precision, with these two models also obtaining the lowest precision rates for this class. The GB model obtained the next highest recall value, 0.696, with precision improved two-fold over the best performing RF model. The highest recall value for burst behaviour was 0.800, obtained by the ANN model; however, the precision value was also the second lowest of all models (0.533). The GB model contributed the best precision value, whilst the LR model performed poorly in both metrics for this class, yielding the lowest class *F*-measure overall (Table 6).

An increase in the class *F*-measure for chafe, burst and headshake classes indicated that they particularly benefitted from the VE technique. Resting was the only class to show a decreased *F*-measure from the VE when compared to the best performing base learners for that class (RF models), however, this difference was small (i.e., 0.977 vs 0.983). The macro-averaged *F*-measure indicated that the VE model improved overall classification above the strongest base learner model (VE: 0.888, GB: 0.856) and showed considerable improvement over the LR model, which performed the weakest of those included after the model validation stage (0.723).

All feature vectors were included in the base learner models. The relative importance of these features varied between the GB and RF models, although both models identified mean ODBA as an important metric (Fig. S1).

### Classifier application

**Fig. 3:**
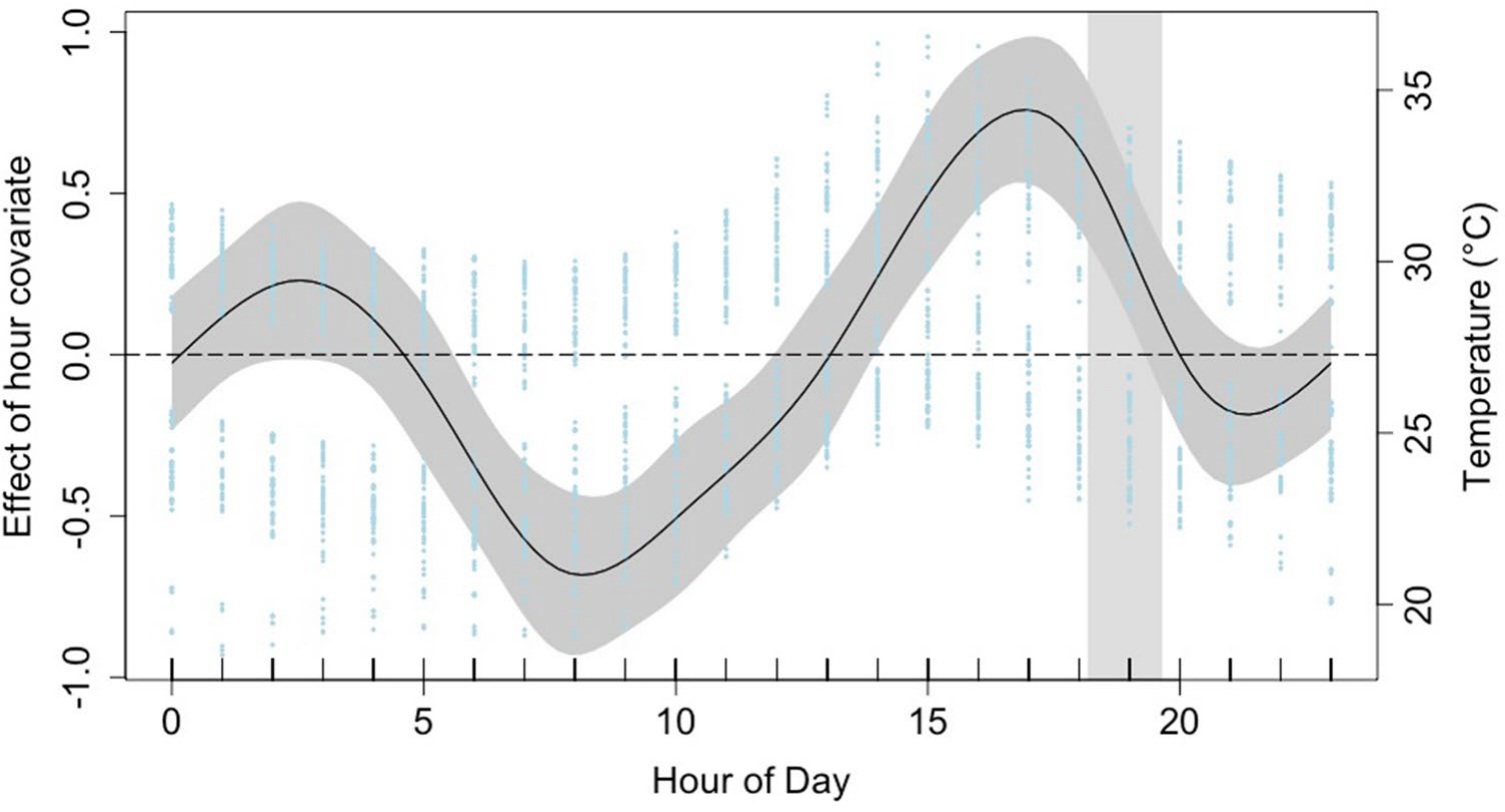
Estimated smoother for the effect of hour of day on the probability of headshaking behaviour occurring by the juvenile lemon shark in Bimini, Bahamas. The lowest and highest probabilities of a headshake occurring are around 0800 and 1700 h, respectively. Estimates are based on final binomial generalized additive mixed model. The solid line is the smoother. Dark grey shaded area surrounding the smoother represent 95% confidence intervals. The light grey shaded area represents the range of sunset times throughout the deployments. The dashed line represents the mean likelihood of a headshaking occurring. The blue dots represent mean hourly temperature (°C), calculated from the temperature sensor in the acceleration data logger (ADL) packages, across all deployments

Behavioural classifications were applied to 2400 h of accelerometry data obtained in the wild (*n* = 18). Headshake predictions were then used to gain insight into temporal dynamics of foraging behaviour. GVIF scores (> 3) revealed collinearity between temperature and season. Temperature was removed as a covariate in favour of season, as all deployments occurred in two distinct seasons and observations suggested feeding increased in the warmer, wet season. The time series included in the GAMM did not show significant auto-correlation and subsequently did not require an auto-correlation structure. Covariates season, tidal phase and time of day were included in the optimal model (Table 7). All covariates were significant in predicting the presence of successful predation events for the juvenile lemon shark in Bimini (Table 8). Presence of hourly headshakes varied, with the dominant peak occurring around 1700 h and a smaller peak around 0230 h (Fig. 3). Headshakes occurred less frequently over high tide and during the dry season (Table 8).

**Table 7:** Log-likelihoods scores for models investigating the occurrence of headshakes in lemon sharks in Bimini, Bahamas

Covariates	Log-likelihood
s(Hour) + factor(Tide) + (Season)	− 1265.815^a^
s(Hour) + factor(Season)	− 1268.462
s(Hour) + factor(Tide)	− 1269.209
s(Hour)	− 1271.833
factor(Tide) + (Season)	− 1300.566
factor(Tide)	− 1304.014
factor(Season)	− 1311.745

^a^Optimal model

**Table 8:** Results of the final binomial generalized additive model investigating the presence of headshaking by lemon sharks in Bimini, Bahamas

Covariate		edf	ref.df	X^2^	*p* value	*R*^2^ (adj.)
Hour		5.903	8	73.82	≤ 0.05	

–	Level	Coefficient	SE	*z* value	**–**	
Tide	Intercept	0.576	0.212	2.722	≤ 0.05	0.0829
	Flood	0.012	0.132	0.090	0.929	
	High	− 0.600	0.149	− 4.035	≤ 0.05	
	Low	0.028	0.144	0.197	0.844	
Season	Dry	− 0.868	0.282	− 3.082	≤ 0.05	

Outcomes of the smoother hour include: covariate, effective degrees of freedom (edf), reference degrees of freedom (ref.df), Chi squared value (*χ*^2^), *p* value. Outcomes of factors include covariate, level, coefficient, standard error (SE), *z* value and *p* value. The overall adjusted *R*^2^ value is also displayed

## Discussion

The main aim of this study was to develop ML models for converting ADL-derived features into behaviours, and to discern which ML classifiers performed best, for prediction of behaviours in the wild. Using the headshake class, we investigated whether the predictions, when considered in the context of relevant abiotic variables, would be suitable for drawing biologically relevant conclusions.

### Voting ensemble classifier

It is important to consider the purpose of the classifier when establishing rare behaviours (i.e., feeding or burst events). If the goal is, as in this case, to investigate patterns of activity, an increased number of false positive predictions assigned to a rare class can obscure patterns in behaviour. For this reason, although the recall of the RF models was better than the other base learners and VE, the lack of precision made this model impractical as a stand-alone classifier, e.g., the RFE model predicted 209% of the actual number of headshakes in the test set. Similarly, increased precision but poor recall, as in the case of the ANN classifier for the headshaking class, may result in a loss of ‘true’ information, and less clarity in behavioural patterns.

Class error output in all models was highest for the two rarest classes—0.26 and 0.30 for headshaking and burst behaviours, respectively in the VE. This is largely due to the comparatively small class sizes, resulting in the misclassification of one event having an overall greater impact on error output. This is a reflection of the extensive time (and subsequent ADL battery, memory capacity and cost) required to obtain data on infrequent behaviours in the lemon shark prohibiting a larger sample size. Such difficulties will vary with model species. Additionally, no headshaking occurred during one of the eight feeding events recorded during captive trials, representing a false negative rate of 12.5%. In the future, obtaining further records of feeding specifically would indicate the accuracy of the current false negative rate associated with this class (which may be related to prey size), whilst generally increasing records of rare behaviours would help overcome the class error output problem and likely improve classification performance by providing more events to train the model. The metrics used to assess ML performance should be considered in instances where correct classification of rare events is of interest. Accuracy is an often-referenced measure but can be misleading in such situations (Valverde-Albacete and Peláez-Moreno 2014).

Both the RF and GB models allow insight into the relative importance of predictor variables (Fig. S1). Overall, the models differed in their choices of important predictors, but agree that mean ODBA plays an important role. The difference in importance may be related to how the models spread the significance of correlated predictors, with GB models concentrating importance in a single variable and RF dispersing the importance across correlated variables (Freeman et al. 2015). ODBA is likely to be crucial in determining resting behaviour, where dynamic body movement ceases. Chafe, burst and prey capture behaviour exhibit increased ODBA values over steady swimming (Fig. 1).

Collecting ground-truthed data in realistic environmental conditions is important. In addition to being more likely to elicit natural behaviours, semi-enclosed pens are subject to ambient abiotic conditions and water movements that can inflate ODBA values obtained during rest periods (Whitney et al. 2010; Lear et al. 2017). Failure to account for these water movements during model training may result in misclassification of data obtained in the wild. Additionally, although not captured during captive trials and therefore not included as a behavioural class in this study, there is the potential for brief moments of gliding during swimming behaviour, which may be classified as resting. Therefore, resting predictions that occur sporadically and are of short duration should be considered with caution. For sharks that glide as part of their activity budget, the addition of vertical velocity as a feature vector may be beneficial for differentiating between resting and gliding. Considering headshakes in conjunction with burst-swimming events may also aid in distinguishing false positive headshakes, as these are likely to occur together as part of foraging behaviour. Burst-swimming events that are not succeeded by headshaking may represent a failed predation attempt or predator avoidance behaviour.

We have demonstrated that classification performance is dependent on the ML method applied, but it can also be affected by the number of classification categories and epoch length (Ladds et al. 2017). Although in this instance, the VE classifier performed better than the constituent base learners, there are some notes of caution for researchers looking to employ this method. First, the base learners employed here are not exhaustive and therefore the ability of this VE classifier to outperform other untested ML methods cannot be indicated. Second, the performance of the VE classifier has not yet been examined outside of our model species or across ontogeny and therefore we cannot attest to its ability to generalize beyond the conditions under which it was developed. Due to the vast range of body movement across the animal kingdom, it is unlikely a single method will provide optimum performance across all species (Ladds et al. 2017). Finally, the ML algorithms employed here do not account for auto-correlation which is expected in chronological acceleration data. Leos-Barajas et al. (2017) advise that whilst this may not matter in instances where the end goal is solely behavioural classification, using the output of such ML classifiers in subsequent statistical steps may render fraudulent results. The results of our study reflect ongoing observations around the Bimini Islands and as such, not accounting for serial-dependence in the classifier development stage does not appear to have impacted the results of our classifier application. However, this may not always be the case and inclusion of an auto-correlation feature vector may be required (e.g., Nathan et al. 2012; Ladds et al. 2017) or alternative models, such as hidden Markov models, which account for temporal dependency could be more applicable (Leos-Barajas et al. 2017; Dhir et al. 2017).

### Classifier application

In this study, we selected headshaking behaviour to investigate whether the behavioural predictions made on wild data yielded biologically relevant results in relation to abiotic variables. During observations of wild and captive sharks, headshaking behaviour for juvenile lemon sharks at Bimini has only been witnessed as part of prey capture and therefore it is considered here as a proxy for successful foraging. Cortés and Gruber (1990) conducted an extensive stomach eversion study on lemon sharks from Bimini and Florida, USA. Using estimated time of consumption, they deemed that feeding in juvenile lemon sharks [43–83.7 cm precaudal length (PCL)] was asynchronous in relation to time of day and tide, and that they were opportunistic feeders. Here, we show that significantly fewer successful predations occurred during the high tide. This supports anecdotal evidence from Bimini, Bahamas, suggesting that feeding occurs more often over the low tides than the high tides (Guttridge 2009; Guttridge et al. 2012). Guttridge (2009) identified that many individuals sought refuge in a mangrove inlet from larger predators able to access the lagoon during high tides, e.g., large lemon sharks (Guttridge et al. 2012) and tiger sharks (*Galeocerdo cuvier*; Hansell et al. 2017). This tidally driven habitat selection is thought to be determined by anti-predator behaviour rather than increased foraging prospects, as only one hunting event was witnessed in the mangrove inlet refuge in more than 70 days of direct observations (Guttridge et al. 2012).

Conversely, Guttridge (2009) documented juvenile lemon sharks moving to more exposed areas during low tides, such as the lagoon, where four predations and 12 foraging-related events (e.g., chasing fish) were witnessed during only 23 days of direct observations. Additionally, prey preference studies conducted at Bimini indicate juvenile lemon sharks feed preferentially both in terms of prey species and prey size, but can feed opportunistically when necessary (Newman et al. 2010, 2011). These contrasting findings indicate that the location of a nursery ground—even within a population—may affect feeding habits of juvenile lemon sharks, warranting further study.

Although studies conducted around the Bimini Islands versus those conducted in a laboratory conflict as to whether the juvenile lemon shark is predominantly nocturnal or crepuscular, rates of movement and metabolic rates are lowest during daylight hours (Morrissey and Gruber 1993; Nixon and Gruber 1988; Sundström et al. 2001). This is reflected in our results, where the incidence of successful predations is lowest during the morning and middle of the day. We found time of day to be a significant covariate affecting successful predations. Most feeding events occurred during early evening, close to sunset; this may be due to decreasing light levels. Sharks possess a reflective layer (tapetum lucidum) in the choroid, behind the retina, which enhances vision in low light conditions (Gardiner et al. 2012). Due to this visual adaptation, Sundström et al. (2001) suggest juvenile lemon sharks might hunt more actively during crepuscular or nocturnal periods, experiencing more frequent success during twilight. Papastamatiou et al. (2015) also found blacktip reef sharks (*Carcharhinus melanopterus*) are more active during the early evening and suggest this may be linked to increased foraging effort when they have a visual advantage over their prey.

Successful predations in the early evening may also be linked to diel temperature fluctuations. The body temperature of a poikilothermic shark, such as the lemon shark, is driven by ambient water temperature. Warmest daily water temperatures are experienced in the North Sound and Bonefish Hole nurseries during mid-afternoon, at ~ 1500 h (DiGirolamo et al. 2012; Fig. 3), approximately two hours before the highest presence of successful predations occur (Fig. 3). Blacktip reef sharks were most active as body temperatures began cooling after reaching their warmest temperatures for the day (Papastamatiou et al. 2015). The authors hypothesised that as predator escape responses scale at a greater rate with temperature than attack rates, blacktip reef sharks may exploit the higher thermal inertia that their body size confers, keeping their body temperature elevated for longer than their prey, increasing chances of successful predation. This may also apply to the juvenile lemon shark.

Significantly fewer incidents of predations occur during the dry season than in the wet season (Table 8), which falls in line with other findings (e.g., juvenile lemon sharks grow faster during the wet season; Gruber unpublished data). Clark (1959) found lemon sharks in semi-captive pens consumed less throughout the colder months, when temperatures fell below 24 °C. During our deployments, ADL temperature loggers recorded a range of 26.3–36.6 °C ($\bar{x} = 30.47$) and 18.5–29.2 °C ($\bar{x} = 24.10$) during the wet and dry season, respectively. It is, therefore, expected that metabolic demands would be higher during the wet season (Lear et al. 2017) and energy intake would need to be augmented to meet these demands, whilst still allowing energy for somatic growth. This may be the result of increased foraging effort and/or greater prey abundance.

A further consideration is the attachment site of ADLs for the behaviour in question. In this case, we were interested in overall behaviour exhibited by juvenile lemon sharks, not only feeding events. Placement of mandible ADLs have been used to successfully identify foraging in marine mammals (e.g., Weddell seals [(*Leptonychotes weddellii*), Naito et al. 2010] and Stellar sea lions [(*Eumetopias jubatus*), Viviant et al. 2010], loggerhead turtles [(*Caretta caretta*), Okuyama et al. 2010] and the common carp [(*Cyprinus* carpio), Makiguchi et al. 2012]). Although the suction-feeding mechanisms differ between ray-finned fishes and elasmobranch fishes (Wilga et al. 2007), the latter study is of particular interest in relation to elasmobranchs that employ suction feeding as their principal feeding mode (e.g., the nurse shark (*Ginglymostoma cirratum*) and whitespotted bamboo sharks (*Chiloscyllium plagiosum*)), which may not be readily distinguished through a dorsally mounted accelerometer.

In this instance, the behavioural classifications were binned as presence/absence by hour, as this was sufficient to demonstrate the application of the classifier to draw ecologically relevant conclusions. It illuminated patterns in the predatory behaviour of juvenile lemon sharks in relation to diel- and tidal cycles, as well as season. ADLs are capable though of providing fine-scale information and the time-window should allow appropriate resolution for the question being addressed. One potential drawback to modelling the presence or absence of headshaking in hourly bins is that multiple prey captures within an hour and a single prey capture event would contribute to the analysis equally. However, this method was selected due to the variation in time spent prey-handling a single item. It may be beneficial for future studies to use mandible accelerometers to address whether there is a correlation between prey size and headshaking duration or intensity and whether prey handling techniques vary with prey type to allow for more quantitative analysis. Accelerometers have been used to identify feeding according to prey type in the white-streaked grouper [(*Epinephelus ongus*); Kawabata et al. 2014] and red-spotted grouper [(*Epinephelus akaara*); Horie et al. 2017] differentiating between shrimp, fish and crab. Although teleosts form most of the juvenile lemon shark diet, they have also been documented with crustaceans, other elasmobranch species [whiptail stingrays (Dasyatidae)], molluscs and annelidas in their stomachs (Newman et al. 2010).

Identification of behaviours exhibited in the wild allows construction of activity budgets. Accelerometer derived ODBA values have proven to be a valuable proxy for energy expenditure for many species, including teleosts (Wright et al. 2014; Metcalfe et al. 2016) and elasmobranchs (Gleiss et al. 2010; Lear et al. 2017). Once this relationship is established, pairing behavioural states with concurrent ODBA values can provide activity specific metabolic rates for deriving time-energy budgets for animals in situ. This was unattainable for aquatic species prior to the development of ADLs and now allows insight into the energetic costs of behavioural decisions, which have implications for fitness. Therefore, the mean daily field metabolic rate will be sensitive to changes in the activity budget (Jodice et al. 2003), which may shift because of human disturbance (e.g., wildlife watching, Constantine et al. 2004; Christiansen et al. 2013; Barnett et al. 2016), natural disturbance (e.g., climate events), seasons (Hanya 2004), habitat quality and food availability (Wauters et al. 1992; Li and Rogers 2004). In future studies, quantitative values of field metabolism (Lear et al. 2017) and relative feeding rates may allow for broad intra-species comparisons across climatic zones and environments with varying anthropogenic disturbance.

In conclusion, this study demonstrates the utility of a voting ensemble ML algorithm and its effectiveness as a classifier for predicting behaviours from accelerometer data. ML techniques are, and will continue to be, increasingly relied upon as accelerometer technology develops and the high information content they can obtain grows. This study indicates why selection of the most appropriate ML algorithm requires careful consideration of classifier application to allow for meaningful subsequent modelling. The precision and recall value for each class predicted by the VE model was not necessarily greater than the base-learners. However, the overall performance was superior by obtaining a balance of good recall, and model precision. This careful classifier development allowed for modelling of a behaviour against abiotic factors, showing that time of day, tidal phase and season are all significant factors in predicting feeding by the lemon shark. In doing so, it has provided empirical evidence that explains observations from numerous studies and has presented insight into the feeding ecology of the juvenile lemon shark.

## Electronic supplementary material

Below is the link to the electronic supplementary material.

Supplementary material 1 (PDF 135 kb)
