# Supplementary material for: Development and application of a machine learning algorithm for classification of elasmobranch behaviour from accelerometry data
Source: Mar Biol. 2018 Mar 8;165(4):62. doi: 10.1007/s00227-018-3318-y (PMC5842499; doi:10.1007/s00227-018-3318-y)
Supplement: Supplementary file 1 — Supplementary material 1 (PDF 135 kb) [file 227_2018_3318_MOESM1_ESM.pdf]

Development and Application of a Machine Learning Algorithm for Classification of Elasmobranch Behaviour from Accelerometry Data

L. R. Brewster[a,b,c,1], J.J. Dale[d], T.L. Guttridge[a], S. H. Gruber[a,e],, A.C. Hansell[f], M. Elliott[b], I. G. Cowx[c], N. M. Whitney[g], A. C. Gleiss[h]

[a] Bimini Biological Field Station Foundation, South Bimini, Bahamas.

[b] Institute of Estuarine & Coastal Studies, University of Hull, HU6 7RX, UK.

[c] Hull International Fisheries Institute, University of Hull, Hull, HU6 7RX, UK.

[d] Department of Biology, Hopkins Marine Station, Stanford University, Pacific Grove, CA 93950, USA

[e] Division of Marine Biology and Fisheries, Rosenstiel School of Marine and Atmospheric Science, 4600 Rickenbacker Causeway, Miami, Florida 33149, USA.

[f] Department of Fisheries Oceanography, School for Marine Science and Technology, University of Massachusetts Dartmouth, 836 South Rodney French Blvd., New Bedford, MA 02719, USA

[g] Anderson Cabot Center for Ocean Life, New England Aquarium, Central Wharf, Boston, MA 02110, USA

[h] Centre For Fish and Fisheries Research, School of Veterinary and Life Sciences, Murdoch University, 90 South Street, Perth, WA 6150, Australia

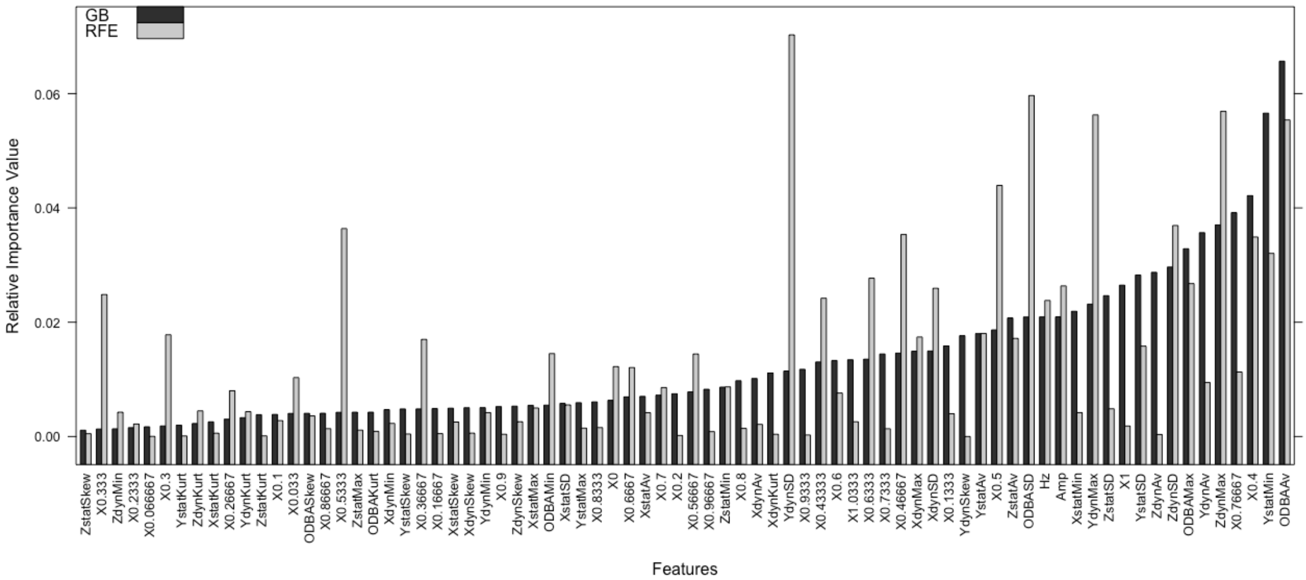

Fig. S1 Feature variable importance for the gradient boosting (GB) model and random forest entropy (RFE) model

[1] Corresponding author: Tel.: +44 (0) 1483 42 74 35; email: lauran.brewster@gmail.com
